# Everyday discrimination among middle-aged and older adults in India: a multilevel cross-sectional analysis from the Longitudinal Ageing Study in India

**DOI:** 10.1038/s41598-026-37790-7

**Published:** 2026-02-14

**Authors:** Ravi Sadhu, Soohyeon Ko, S. V. Subramanian, Rockli Kim

**Affiliations:** 1 Department of Social and Behavioral Sciences, Harvard T.H. Chan School of Public Health, Boston, MA USA; 2 Harvard Center for Population and Development Studies, Cambridge, MA USA; 3 Division of Health Policy and Management, College of Health Science, Korea University, Seoul, Republic of Korea; 4 Interdisciplinary Program in Precision Public Health, Department of Public Health Sciences, Graduate School of Korea University, Seoul, Republic of Korea

**Keywords:** Everyday discrimination, Geographic variation, Correlates, Multilevel, Adults, India, Epidemiology, Risk factors

## Abstract

Everyday discrimination (ED) has adverse effects on health and well-being. This study highlights understudied trends in the distribution, correlates, and geographic variation of ED among Indian adults aged 45 and above. The analysis of 61,722 participants in the Longitudinal Ageing Study in India (2017-18) revealed significant state/union territory (UT)-level variation. While Nagaland, Tripura, Mizoram, and Lakshadweep had comparatively lower ED scores than the national average, Jammu and Kashmir, Delhi, Chhattisgarh, and Karnataka had higher scores. Additionally, using multilevel negative binomial regression, we found that men, non-married adults, migrant residents, and adults in rural areas had higher ED rates. In general, with increases in education level and household monthly per capita income, there was a reduction in ED rates. Notably, adults with a functional disability (Incident Rate Ratio (IRR) = 1.43 [95% confidence interval: 1.32, 1.55]) and physical or mental impairment (IRR = 2.15 [1.88, 2.45]) had significantly higher ED rates than those without. We also partitioned the geographic variation in ED and found that more geographic variance was explained by the community (village/ward) level than by the state/UT level, accounting for roughly 60% and 40% of the variation across models, respectively. Our findings suggest that community-based contextual factors necessitate further research.

## Introduction

Discrimination is a pervasive psychosocial force that manifests at various levels—interpersonal, institutional, and structural—and has tangible implications for health and well-being, particularly when it is perceived or experienced^1–3^. These can include heightened stress reactions, maladaptive coping behaviors such as increased substance use, and poorer physical and mental health outcomes^3–6^. Everyday discrimination—capturing “chronic, routine, and relatively minor experiences of unfair treatment”^7^—has emerged as the dominant indicator in the public health literature for measuring perceived discrimination and quantifying the psychosomatic health effects when these experiences are internalized^3,5^. Though the literature on everyday discrimination (ED) and ill health in India is nascent, nationally representative studies from the Longitudinal Ageing Study in India 2017-18 (LASI) have shown that ED is positively associated with physical frailty, depressive symptoms, poorer cognition, worse sleep, and loneliness^8–14^. Additionally, ED has been found to mediate the relationship between low perceived socioeconomic status and frailty and to drive urban-rural and caste-based differences in depression among older Indian adults^8,10,12^. Given its association with adverse health outcomes, it is crucial to examine how ED itself is patterned along sociodemographic and geographic lines among adults nationally.

Analogous to the case in most countries worldwide, numerous forms of inequality and discrimination are entrenched in India’s social fabric. As well-established, caste, gender, religious, regional, and class-based hierarchies reinforce one another to influence the differential treatment of large sections of Indian society^15–18^. Smaller quantitative and qualitative studies have highlighted the experiences of vulnerable populations. As examples, tuberculosis patients in Kolkata had a high rate of reporting ED from neighbors and colleagues, with a pronounced rate among women and older adults^19^. Additionally, transmasculine men in Mumbai and Chennai also reported experiencing heightened ED^20^. In rural Gujarat, a study of 170 pregnant women found that over 68% of women reported ED, with lower-caste women reporting higher scores^21^. At the national level, four LASI studies highlight the correlates of ED among adults aged 60 and above^15,22–24^. Overall, those with poor health, lower education, and lower subjective socioeconomic status were more likely to report higher general ED and age-related ED^15,22–24^. Intersectional patterns are apparent in ED, with low-caste, low-income women being especially likely to report ED^15^.

While the current landscape is informative and enlightening, the literature at the national level is restricted to older adults alone. Additionally, geography is underexplored in relation to ED in India. The norms regarding the forms, extent, and expression of discrimination rooted in political hierarchies, class, caste, gender, and religious dynamics differ from region to region in India^25^. Relatedly, human development metrics—literacy, urbanicity, infrastructure, and economic inequality—differ across and within states^26–28^, and may influence patterns in ED. Lastly, from a historical perspective, social movements have evolved differently across the country, shaping distinct collective identities and awareness of discrimination against groups such as women, Dalits, peasants, Adivasis, and farmers^29–31^. Both compositional (individual characteristics such as gender and socioeconomic status) and contextual (group-level characteristics such as government policies and community norms) factors shape various health and social outcomes, including ED^32,33^. Considering India’s staggering diversity, a focus on geography facilitates a more coherent and unified understanding of whether compositional or contextual factors are more influential in explaining ED variation and at what level (state, regional, district, or community). Ours is a novel effort to analyze the spatial variation in ED among Indian adults aged 45 and above. We examine the following questions: (1) Does ED differ by state/UTs? (2) Accounting for geographic variation, what demographic, socioeconomic, and health factors are correlated with ED? (3) Which geographic level (state/UT or community, i.e. rural village/urban ward) explains a more significant proportion of the geographic variation of ED in India? and (4) To what degree do common demographic, socioeconomic, and health factors explain the geographic variation in ED?

## Methods

### Data source and study sample

The Longitudinal Ageing Study inIndia (LASI) is a nationally representative study of 73,396 Indian adults aged 45 and above, along with their spouses. Wave 1 of LASI was conducted from 2017 to 2018 across India’s states and union territories (UTs), excluding Sikkim, for which data were collected from 2020 to 2021. A multistage stratified sampling design was utilized to recruit participants^34^. In each state/UT, the first stage involved selecting primary sampling units (PSUs), which are sub-districts (*tehsils* or *taluks*). The secondary stage involved selecting secondary sampling units (SSUs). SSUs were villages in rural areas and wards in urban areas; hereafter, they are collectively referred to as “communities.” We used the Wave 1 dataset from the Indian Institute of Population Sciences, which is publicly accessible. LASI Wave 1 was approved by the Indian Council for Medical Research, the Ministry of Health and Family Welfare, and the ethical boards of participating institutions and universities. Respondents gave informed consent to participate^34^. Since we analyzed publicly available and de-identified secondary data, our study was exempt from a full institutional review.

### Correlates and outcome

Based on a literature review of previous studies on perceived discrimination in India, we selected 12 explanatory variables as potential correlates relevant to the Indian context^15,19–24^. These included seven demographic factors, namely age group (45–54 years, 55–64 years, 65–74 years, and 75 years and above), gender (men/women), marital status (married/widowed/other [divorced, separated, in a live-in relationship, or never married]), religion (Hindu/Muslim/Christian/Other [no religion, Sikh, Parsi, Jew, and other]), caste (Upper Castes or Other Castes/Scheduled Castes/Scheduled Tribes/Other Backward Classes), migration status (lived in the current area of residence for their whole life/has lived in their current location for ten years or more/ has lived in their current location for less than ten years), and place of residence (rural/urban). We also considered three socioeconomic factors, namely education level determined by the number of years of formal schooling (no schooling/1–5 years/6–9 years/10 or more years), household monthly per capita expenditure quintile (poorest/poor/middle/richer/richest), and working status (currently working/currently not working/never worked for more than three months in their lifetime). Last, we were interested in the association between health status and ED. We therefore considered two health factors: functional disability (yes/no) and impairment (yes/no). To determine functional disability status, we aggregated and scored activities of daily living (ADL) and instrumental activities of daily living (IADL). A respondent with at least one limitation of ADLs or IADLs was classified as having a functional disability. Impairment status was classified based on their response to the following survey question: Do you have any form of physical or mental impairment? (yes/no). Impairments were wide-ranging, including those affecting the lower or upper body, intellectual, cognitive, or learning functions, as well as hearing, visual, or speech challenges.

LASI employed a modified six-item version of the Everyday Discrimination Scale^35^, which we used to construct our outcome. Respondents were asked if any of the following happened to them in their day-to-day life: (1) You are treated with less courtesy or respect than other people; (2) You receive poorer service than other people at restaurants or stores; (3) People act as if they think you are not smart; (4) People act as if they are afraid of you; (5) You are threatened or harassed; and (6) You receive poorer service or treatment than other people from doctors or hospitals. Participants were asked to rank the frequency of these six things happening to them, ranging from 1 (almost every day) to 6 (never). Respondents with missing data for any of the six items were excluded. Acknowledging that ED includes the perception of mistreatment attributed explicitly to group membership or a particular characteristic^36^, we only included respondents who identified one or more reasons for their experiences of ED. These primarily included age, gender, religion, caste, weight, disability, physical attributes, and financial status. Additionally, respondents who qualitatively identified another reason for their mistreatment—health status (physical illness or mental health problems), socioeconomic status (profession, education, or type of residence), marital status, and nationality—were included. Rather than focusing on a specific domain of discrimination (age, gender, financial status, etc.), our outcome captures general everyday discrimination.

**Fig. 1:**
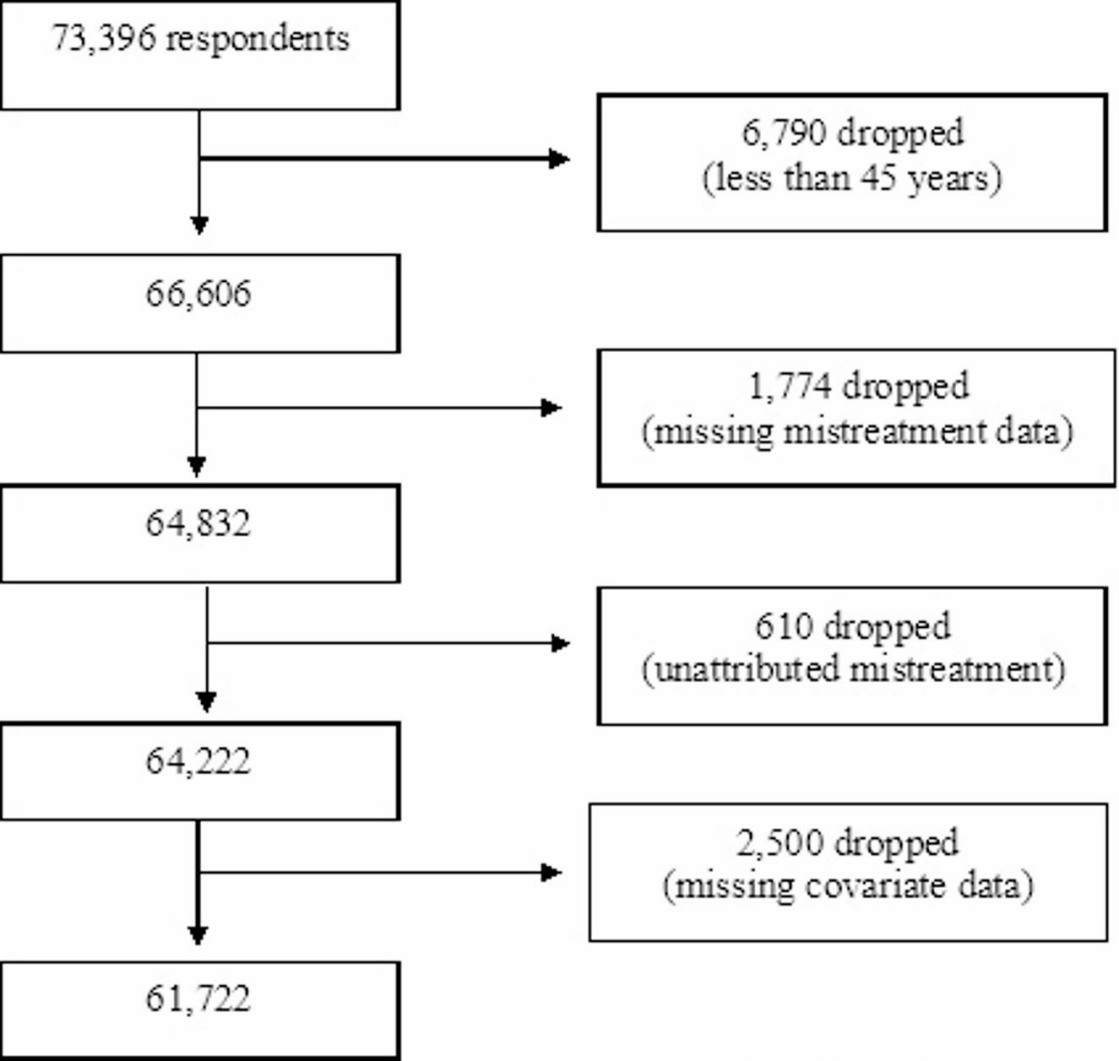
Flowchart of the selection of the study sample.

Overall, we excluded (1) participants under the age of 45, (2) participants with missing data for instances of everyday discrimination and its attributable cause or causes, and (3) participants with missing demographic, socioeconomic, and health covariate data. Our final sample consisted of 61,722 adults (Fig. 1). In our final sample, the Cronbach’s alpha score among the six ED items was 0.874, indicating high reliability. For each of six items, we reverse-coded responses from 0 (never) to 5 (almost every day). We then summed the scores across the six items to obtain an aggregate score ranging from 0 to 30, with higher scores indicating greater ED.

### Statistical analysis

To highlight the distribution of ED, we computed the weighted mean and standard deviation by each subgroup for the 12 demographic, socioeconomic, and health correlates, as well as for all states and UTs in India. We also reported the proportion of respondents who reported experiencing some form of discrimination, i.e., an EDS score greater than zero. For all descriptive analyses, we used sampling weights to account for the complex survey design effects of LASI.

Using multilevel modeling, we evaluated the association between the 12 correlates and ED (continuous score of 0–30). We adopted a three-level multilevel mixed-effects model, with adults (level 1) nested within communities (level 2), which were in turn nested within states/UTs (level 3). Over 85% of the sample reported not experiencing any form of ED, resulting in a significant skew in the ED outcome. We employed a negative binomial regression model, which can account for overdispersion in count data^37^. Starting with a null model, we incrementally added demographic, socioeconomic, and health correlates in three additional models. Our final model included all three types of correlates. We also computed the variance partitioning coefficient (VPC) to gauge the comparative importance of the community and state/UT levels in explaining the geographic variation in ED, following the methods previously described for calculating VPC^27,38^. We performed all analyses using Stata 19.5.

## Results

Our sample consisted of 61,722 adults from 2,249 communities across all 36 Indian states/UTs. Over 89% of the sample was under 75 years of age, and 54% were women (Table 1). Most adults were married to a living spouse, were Hindu, and lived in rural areas. Nearly 47% of the sample reported having “Other Backward Classes” (OBC) caste status. Additionally, 57% had migrated to a different area to live at some point in their lives, and fewer than half of the participants were currently working. 51% of the sample had never received schooling, and just under 18% reported attending school for ten years or more. The sample was nearly evenly divided among the five wealth quintiles. Almost 40% and 8% of respondents reported having a functional disability and some mental or physical impairment, respectively.

**Table 1:** The weighted distribution of everyday discrimination for India and 36 States and union territories, Longitudinal Ageing Study inf India (Wave 1, 2017-18).

	Sample Distribution		Everyday Discrimination	
	*n*	%	Mean (SD) for score ≥ 0	Weighted % [95% CI] for score ≥ 1
Total	61,722		1.0 (3.1)	16.8 [15.7, 18.0]
Age group				
45–54 years	22,800	35.3	1.0 (3.2)	16.8 [14.7, 19.1]
55–64 years	19,098	30.0	0.9 (3.1)	16.4 [15.3, 17.6]
65–74 years	13,668	23.9	1.0 (2.9)	17.3 [15.5, 19.2]
75 + years	6,156	10.8	1.0 (2.9)	17.2 [15.3, 19.1]
Gender				
Men	28,688	45.8	1.0 (3.1)	16.9 [15.6, 18.3]
Women	33,034	54.2	1.0 (3.0)	16.7 [15.6, 17.9]
Marital status				
Married	46,162	73.7	0.9 (3.0)	16.2 [15.0, 17.6]
Widowed	13,526	23.5	1.0 (3.1)	18.5 [16.8, 20.3]
Other	2,034	2.9	1.2 (3.6)	18.0 [13.4, 23.9]
Religion				
Hindu	45,921	83.4	1.0 (2.9)	17.0 [15.9, 18.1]
Muslim	6,494	10.0	1.1 (3.1)	19.0 [13.1, 26.9]
Christian	6,105	3.0	0.9 (5.5)	13.6 [9.6, 18.9]
Other	3,202	3.6	0.6 (3.0)	10.7 [8.4, 13.4]
Caste				
Upper Caste	15,648	24.9	0.8 (3.0)	13.4 [12.1, 14.8]
Scheduled Caste (SC)	10,612	19.8	1.1 (3.0)	18.8 [17.0, 20.9]
Scheduled Tribe (ST)	11,244	8.9	0.9 (4.6)	16.0 [14.0, 18.1]
Other Backward Classes (OBC)	24,218	46.5	1.0 (2.7)	18.0 [16.1, 20.0]
Migration status				
Lived for whole life	26,815	42.6	0.9 (3.0)	16.2 [14.9, 17.7]
Lived for 10 years or more	32,350	54.0	1.0 (3.1)	16.8 [15.8, 17.9]
Lived for less than 10 years	2,557	3.4	1.6 (4.1)	24.6 [16.4, 35.2]
Place of residence				
Rural	40,383	69.3	1.0 (2.9)	17.5 [16.3, 18.7]
Urban	21,339	30.7	1.0 (3.3)	15.4 [12.9, 18.4]
Education level				
No schooling	29,036	50.7	1.1 (3.1)	19.1 [17.8, 20.5]
1–5 years of schooling	11,336	17.4	0.9 (3.0)	16.5 [15.2, 18.0]
6–9 years of schooling	9,780	14.0	0.8 (3.0)	14.5 [11.9, 17.5]
10 years of schooling or more	11,570	17.8	0.7 (2.9)	12.5 [10.4, 15.1]
Household monthly per capita expenditure				
Poorest	12,311	21.1	1.0 (2.9)	17.7 [16.2, 19.3]
Poor	12,437	21.1	1.0 (2.9)	16.3 [14.9, 17.7]
Middle	12,391	20.3	0.9 (3.0)	15.8 [14.2, 17.4]
Richer	12,448	19.4	0.9 (2.9)	16.3 [14.4, 18.4]
Richest	12,135	18.0	1.1 (3.6)	18.3 [14.6, 22.7]
Working status				
Currently Working	28,564	46.7	0.9 (2.9)	17.5 [16.1, 19.0]
Currently Not Working	16,073	27.2	1.2 (3.3)	19.7 [18.0, 21.5]
Never Worked In Lifetime	17,085	26.2	0.8 (3.0)	12.7 [11.5, 14.1]
Functional disability				
No	39,577	60.1	0.9 (3.3)	14.3 [13.2, 15.5]
Yes	22,145	39.9	0.9 (2.6)	20.7 [19.1, 22.3]
Physical or mental impairment				
No	57,535	92.3	0.9 (3.0)	15.6 [14.6, 16.7]
Yes	4,187	7.7	2.0 (3.9)	31.4 [27.1, 36.1]

Comparisons of average ED scores across subgroups revealed few substantive differences, likely attributable to the pronounced skew of the ED scores towards zero (Table 1). Overall, the national average ED score was 1.0 (standard deviation: 3.1). The differences between average scores comparing adults from the “Other” religious group and Hindu adults, as well as adults with 10 years of schooling or more compared to adults with no formal schooling, both corresponded to 0.4. Adults who had migrated elsewhere within the past 10 years reported average ED scores that were 0.7 points higher than those of adults who had never moved. Most notably, there was a difference of 1.1 in average ED scores between adults with and without physical or mental impairments. We also compared the proportion of adults who report some form of ED (ED score greater than 0), which was 16.8% at the national level [95% confidence interval: 15.7, 18.0]. The proportion of adults did not differ substantially across age groups, gender, marital status, place of residence, or household monthly per capita expenditure, ranging from 16% to 20% (Table 1). Those belonging to the “Other” religions, upper castes, and who had never worked during their lifetime reported lower rates of experiencing some ED (10.7%, 13.4%, and 12.7% respectively) in the religion, caste, and working status categories. There was an 8.4% point difference in reporting ED between those who had migrated to a new area less than ten years ago and those who had never moved out of their home. Notably, as the education level increased, ED decreased. Roughly one in five adults with a functional disability and one in three adults with some mental or physical impairment reported experiencing ED (Table 1).

We found significant geographic variation in ED comparing states and UTs (Table 2). Average ED scores ranged from 0.0 (0.6) in Nagaland to 2.3 (7.8) in Jammu and Kashmir. Scores were equal to or higher than 2.0 in three states/UTs (Chhattisgarh, Delhi, and Jammu and Kashmir) and equal to or higher than 1.5 in 6 states/UTs (Chhattisgarh, Delhi, Jammu and Kashmir, Karnataka, Madhya Pradesh, Puducherry, and Rajasthan). Addressing the proportion of adults who reported experiencing some form of ED, some of the lowest ED rates were in the Northeastern states: 0.5% [0.2, 1.4] in Nagaland, 2.6% [1.3, 5.4] in Tripura, and 3.4% [1.8, 6.3] in Mizoram (Table 2). However, Arunachal Pradesh, with a prevalence of 23.2% [16.5, 31.7], was an exception. Additionally, Odisha and Puducherry had a low proportion of adults reporting ED, 3.5% [2.5, 4.8] and 3.6% [1.7, 7.3], respectively. In seven states (Arunachal Pradesh, Chhattisgarh, Jammu and Kashmir, Karnataka, Madhya Pradesh, Telangana, and Uttar Pradesh) and the UT of Delhi, the proportions exceeded 20%. The highest were in Chhattisgarh and Karnataka, where nearly 31% of adults reported facing ED.

**Table 2:** The weighted distribution of everyday discrimination for India and 36 states and union territories, Longitudinal Ageing Study in India (Wave 1, 2017–18).

State/ UT	Location	Mean (SD) for score ≥ 0	Weighted % [95% CI] for score ≥ 1
India		1.0 (3.1)	16.8 [15.7, 18.0]
States			
Jammu and Kashmir	North	2.3 (7.8)	22.4 [17.9, 29.6]
Haryana	North	0.6 (2.7)	12.5 [10.8, 14.6]
Himachal Pradesh	North	0.4 (2.4)	10.2 [7.2, 14.2]
Punjab	North	0.2 (1.5)	5.7 [3.8, 8.5]
Rajasthan	North	1.6 (4.5)	13.2 [11.0, 15.7]
Uttarakhand	North	0.3 (2.6)	5.7 [4.1, 8.5]
Arunachal Pradesh	Northeast	1.1 (8.1)	23.2 [16.5, 31.7]
Assam	Northeast	0.4 (2.4)	8.2 [6.0, 11.0]
Manipur	Northeast	0.4 (4.3)	10.8 [7.5, 15.4]
Meghalaya	Northeast	0.1 (2.6)	3.9 [2.4, 6.4]
Mizoram	Northeast	0.1 (3.1)	3.4 [1.8, 6.3]
Nagaland	Northeast	0.0 (0.6)	0.5 [0.2, 1.4]
Tripura	Northeast	0.1 (0.9)	2.6 [1.3, 5.4]
Bihar	East	0.7 (1.7)	17.2 [13.2, 22.0]
Jharkhand	East	0.5 (2.4)	9.8 [7.2, 13.3]
Odisha	East	0.2 (1.3)	3.5 [2.5, 4.8]
Sikkim	East	1.2 (21.5)	19.5 [13.1, 28.0]
West Bengal	East	0.5 (1.3)	13.3 [9.7, 17.9]
Chhattisgarh	Central	2.0 (5.4)	31.4 [27.9, 35.2]
Madhya Pradesh	Central	1.6 (2.9)	25.9 [20.6, 32.0]
Uttar Pradesh	Central	1.4 (2.6)	22.1 [19.6, 24.8]
Gujarat	West	0.5 (1.2)	13.8 [11.6, 16.3]
Maharashtra	West	0.5 (1.3)	11.5 [9.1, 14.5]
Andhra Pradesh	South	1.4 (3.8)	16.0 [12.3, 20.5]
Goa	South	0.9 (10.7)	15.0 [11.5, 19.3]
Karnataka	South	1.7 (2.1)	31.6 [23.7, 40.8]
Kerala	South	0.7 (4.0)	10.9 [7.5, 15.6]
Tamil Nadu	South	0.8 (2.2)	14.4 [11.8, 17.5]
Telangana	South	1.3 (4.2)	22.1 [18.3, 26.3]
Union Territories (UTs)			
Chandigarh	North	0.6 (11.6)	13.1 [9.9, 17.1]
Delhi	North	2.3 (6.5)	24.6 [19.0, 31.3]
Dadra and Nagar Haveli	West	0.5 (14.6)	16.2 [12.6, 20.6]
Daman and Diu	West	0.6 (18.2)	16.6 [13.7, 20.0]
Andaman and Nicobar	South	0.5 (20.2)	9.2 [5.6, 14.8]
Lakshadweep	South	0.1 (50.6)	3.6 [1.7, 7.3]
Puducherry	South	1.5 (16.7)	15.4 [11.1, 21.0]

We determined associations between demographic, socioeconomic, and health factors and ED by incident rate ratios (Table 3). Holding all other factors constant (as in all remaining interpretations), women had a 16% lower rate of reporting ED than men (Incident Rate Ratio (IRR) = 0.84 [95% confidence interval: 0.77, 0.92]). We also found that married adults had a lower rate of ED than widowed adults or those who were neither married nor widowed (i.e., divorced, separated, in a live-in relationship, or never married). Religion and caste were also salient correlates, with Christian adults having a 25% lower rate of reporting ED than Hindu adults (IRR = 0.75 [0.61, 0.92]), and adults from SCs having a 29% higher rate of ED than their upper caste counterparts (IRR = 1.29 [1.14, 1.46]). Additionally, urban residents had a 27% lower rate of reporting ED than rural residents (IRR = 0.73 [0.62, 0.86]).

**Table 3:** Multilevel mixed-effects negative binomial regression analysis of the associations between demographic, socioeconomic, and health factors and everyday discrimination, Longitudinal Ageing Study inIndia (Wave 1, 2017–18).

	Incident Rate Ratio	95% CI	*p*-value
Age group			
45–54 years	ref	ref	ref
55–64 years	0.96	[0.88, 1.04]	0.306
65–74 years	0.92	[0.83, 1.01]	0.093
75+ years	0.88	[0.77, 1.02]	0.083
Gender			
Men	ref	ref	ref
Women	0.84	[0.77, 0.92]	< 0.001
Marital status			
Married	ref	ref	ref
Widowed	1.21	[1.11, 1.33]	< 0.001
Other	1.75	[1.43, 2.14]	< 0.001
Religion			
Hindu	ref	ref	ref
Muslim	0.86	[0.73, 1.02]	0.077
Christian	0.75	[0.61, 0.92]	0.005
Other	0.81	[0.65, 1.01]	0.057
Caste			
Upper Caste	ref	ref	ref
Scheduled Caste (SC)	1.29	[1.14, 1.46]	< 0.001
Scheduled Tribe (ST)	1.14	[0.96, 1.35]	0.132
Other Backward Classes (OBC)	1.03	[0.93, 1.15]	0.548
Migration status			
Lived for whole life	ref	ref	ref
Lived for 10 years or more	1.12	[1.03, 1.22]	0.010
Lived for less than 10 years	1.60	[1.34, 1.92]	< 0.001
Place of residence			
Rural	ref	ref	ref
Urban	0.73	[0.62, 0.86]	< 0.001
Education level			
No schooling	ref	ref	ref
1–5 years of schooling	0.96	[0.87, 1.06]	0.421
6–9 years of schooling	0.81	[0.73, 0.91]	< 0.001
10 years of schooling or more	0.69	[0.61, 0.78]	< 0.001
Household monthly per capita expenditure			
Poorest	ref	ref	ref
Poor	0.91	[0.81, 1.01]	0.074
Middle	0.89	[0.79, 1.00]	0.042
Richer	0.88	[0.79, 0.99]	0.035
Richest	0.92	[0.82, 1.04]	0.200
Working status			
Currently Working	ref	ref	ref
Currently Not Working	1.04	[0.95, 1.14]	0.345
Never worked in lifetime	0.91	[0.82, 1.01]	0.087
Functional disability			
No	ref	ref	ref
Yes	1.43	[1.32, 1.55]	< 0.001
Physical or mental impairment			
No	ref	ref	ref
Yes	2.15	[1.88, 2.45]	< 0.001
Intercept	0.27	[0.18, 0.39]	< 0.001

Notably, a graded pattern in ED was evident for migration status, education level, and household monthly per capita expenditure (Table 3). Migrants who have lived in the area for ten years or longer and for less than ten years had 12% (IRR = 1.12 [1.03, 1.22]) and 60% (IRR = 1.60 [1.34, 1.92]) higher rates of ED relative to those of non-migrants, respectively. Additionally, as education levels increased, the reporting of ED decreased; adults who had ten years of schooling or more had a 31% lower rate of reporting ED compared to participants with no formal schooling (IRR = 0.69 [0.61, 0.78]). Additionally, adults in the “middle” (IRR = 0.89 [0.79, 1.00]) and “richer” (IRR = 0.88 [0.79, 1.00]) household per capita expenditure quintiles had lower ED rates than those in the “poorest” quintile. Finally, salient correlates of ED also included health factors (Table 3). Adults with functional disability (IRR = 1.43 [1.32, 1.55]) and some form of mental or physical impairment had significantly higher rates of reporting facing ED (IRR = 2.15 [1.88, 2.45]).

Table 4 shows the variance estimates (log rate units) and the 95% confidence intervals computed from unadjusted and adjusted multilevel models at two levels: state/UT and community. In the null model (Model 1), the variance in ED was estimated at 1.57 [1.00, 2.47] at the state/UT level and 2.23 [2.02, 2.46] at the community level (Table 4). Across all models, a higher proportion of geographic variance in ED was attributable to communities (between 58% and 61%) than to states/UTs (between 39% and 42%). Overall, demographic, socioeconomic, and health factors explained a small percentage of between-state/UT and between-community variance. Adding demographic factors (Model 1), socioeconomic and demographic factors (Model 2), and health, socioeconomic, and demographic factors (Model 3) incrementally increased the explained variance in ED between-states/UTs: 0.6%, 3.5%, and 6.3%, respectively. In contrast, adding health factors slightly reduced the between-community variance explained (from 1.6% in Model 3 to 0.8% in Model 4).

**Table 4:** Variance estimates in log-rate scale [95% confidence interval] and variance (%) explained in everyday discrimination at state/UT and community levels from multilevel mixed-effects negative binomial regression analyses, Longitudinal Ageing Study in India (Wave 1, 2017–18).

	Model 1 *	Model 2 **	Model 3 ***	Model 4 ****
Level 3: States/UTs (n = 36)				
Variance estimate (95% CI)	1.57 [1.00, 2.47]	1.56 [0.98, 2.47]	1.51 [0.95, 2.40]	1.47 [0.92, 2.33]
VPC (%)	41.2%	41.5%	40.8%	39.9%
Variance explained (%)		0.6%	3.5%	6.3%
Level 2: Communities (n = 2,429)				
Variance estimate (95% CI)	2.23 [2.02, 2.46]	2.19 [1.99, 2.42]	2.20 [2.00, 2.42]	2.21 [2.01, 2.44]
VPC (%)	58.8%	58.5%	59.2%	60.1%
Variance explained (%)		1.7%	1.6%	0.8%

*Null Model.

**Adjusts for demographic factors (age group, gender, marital status, religion, caste, migration status, and place of residence).

***Adjusts for demographic (age group, gender, marital status, religion, caste, migration status, and place of residence) and socioeconomic factors (education level, household monthly per capita expenditure, and working status).

****Adjusts for demographic (age group, gender, marital status, religion, caste, migration status, and place of residence), socioeconomic (education level, household monthly per capita expenditure, and working status), and health factors (functional disability and mental or physical or mental impairment).

## Discussion

Four insights emerge from our findings. First, compared to the national estimates, there was considerable variation in ED across states and UTs. The lowest ED was found in Nagaland, Tripura, Mizoram, Meghalaya, and Lakshadweep. The fact that many of these states are in Northeast India might be significant, though Arunachal Pradesh (also in the region) had a higher ED score. Notably, these states stand out considering that Nagaland, Mizoram, and Meghalaya are Christian-majority states, while Lakshadweep is a Muslim majority territory^39^. On the other hand, the leading states/UTs with high ED included Jammu & Kashmir, Delhi, Chhattisgarh, and Karnataka. As such, no simplistic generalizations can be made in the ED patterns we observe along the lines of the extent of interstate and intrastate socioeconomic inequality^40,41^. Various contextual factors likely influence geographic variation in ED at the state/UT level, such as state size, overall ethnoreligious composition, demographic patterns related to in-migration across and within states, languages spoken, degree of urbanization, literacy levels, the proportion of SC/ST communities, and regional identity politics. Further research must explore relevant factors that can explain the trends we find.

Second, our findings indicate that some demographic, socioeconomic, and health correlates are significantly related to ED upon accounting for geographic variation. We found that women had lower rates of reporting ED compared to men. There may be gendered patterns in the exposure to and, potentially, perceptions of inequality and discrimination inside and outside the home, partly due to women’s lower public participation in India than men, especially among older adults^42–44^. Also, unmarried, separated, divorced, or cohabiting adults had higher ED rates. In the absence of the privileges of being married, adults can be exposed to discrimination and exclusion in the form of lack of support from social networks, isolation or loneliness, economic vulnerability, and reduced social capital^45,46^. Surprisingly, adults from the ST and OBC caste groups did not have higher ED rates, and neither did Muslim adults. In fact, we found that Christian adults had lower ED rates compared to their Hindu counterparts. Additionally, migrants, especially those who migrated within ten years, were more likely to report ED. Internal migrants in India, particularly poorer migrants seeking better work opportunities, face numerous challenges, including social hostility, labor market exploitation, and institutional exclusion and marginalization^47–49^. These factors likely contribute to their high ED. Like Chakraborty & Kundu^15^, we, too, observed that education level is a protective factor against ED, as was household monthly per capita expenditure. Another protective factor was urban residence, despite stark segregation and discrimination in urban areas influenced by caste and class^50,51^. Finally, adults with functional disability or impairment reported higher ED, in line with previous studies on the connection between health status and ED^19,22,24^. ED might compound the various social and economic challenges adults with disabilities and chronic conditions already face in India, including elder abuse and loneliness^45,52^.

Third, we found that a higher proportion of geographic variance in ED was attributable to the community level compared to the state/UT level. Previous studies have demonstrated the importance of the neighborhood level (villages and urban wards/blocks) in capturing the variation of different types of inequality in India. Addressing economic disparities, Kim and colleagues found that villages accounted for 12% of the variation of poverty across the country^27^. Additionally, Mohanty and Vasishtha found that census enumeration blocks (equivalent to wards in our study) explained nearly 18% of the variation of multidimensional poverty—an aggregate measure including education, health, standard of living, and housing—in urban India^53^. Several studies have also demonstrated that socioeconomic status and social capital significantly affect health at the neighborhood level in India^54–56^. Our evidence indicates that perceived discrimination, which is a type of social inequality, is also relevant at the community level. Various compositional and contextual factors might contribute to the community variation in ED. The former includes community differences in ethnic, religious, and caste compositions and community-wide wealth and education levels. The latter may consist of enforced SES and caste-based spatial segregation, subregional attitudes towards migrants and adults with disabilities, and discrimination in schools, workplaces, and hospitals. Fourth, our multilevel model did not substantially explain the geographic variation of ED at both the state/UT and the community levels. Though we included a comprehensive range of individual-level correlates in our regression analysis, this was not an all-inclusive list. Several unaccounted factors are likely influential correlates of ED. These include occupation, language, skin color, and physical appearance. Additionally, various higher-level contextual and compositional factors we could not account for, many of which are outlined earlier in this paragraph, may drive geographic ED trends among adults.

Several limitations in our approach must be considered while interpreting our findings. First, we used cross-sectional data; therefore, all the relationships discussed are associative, not causal. Also, ED is a subjective and self-reported measure. What individuals perceive or consider as discrimination or mistreatment may differ, influenced by various factors such as their subjective socioeconomic position, power dynamics at play, and the extent to which unfair treatment may be normalized in their community and regionally^57^. Relatedly, adults from specific social groups might have underreported the frequency and magnitude of everyday discrimination, leading to minimization bias^58^. We hypothesize that this may have been the case for ED among women, adults from lower caste groups and Muslims. Six items of the modified EDS used in LASI may not have fully captured respondents’ discriminatory experiences and may underestimate perceptions of discrimination. Additionally, it must be noted that some correlates, namely health status, have a bidirectional relationship with ED, which may have led to biased estimates. Though our approach’s strength was adjusting for geographic variation, we failed to include variation at the district level, as data were unavailable. The district level also likely accounts for the geographic variation in ED and warrants further attention.

## Conclusion and implications

In this study, we make three fundamental efforts to advance the evidence base on the sociospatial distribution of ED among middle-aged and older adults in India. First, we highlight the heterogeneous distribution of ED across 36 of India’s states and union territories. Second, we apply multilevel modeling to examine the association between 12 demographic, socioeconomic, and health correlates of ED. Third, we partition the geographic variation in ED and present the importance of the community level (village/ward) in shaping ED trends in India. Our findings indicate that there are more unknowns than knowns in explaining trends in the geographic and social distribution of ED among adults in India. Research efforts must assess subgroup differences to validate whether cross-group comparisons of ED can be made using the Everyday Discrimination Scale for Indian adults from diverse caste, religious, and economic backgrounds, as previous research from the United States has highlighted general challenges with comparing ED scores across social subgroups^36,59^. Studies must also investigate whether language plays a role in interpreting questions assessing ED in India. The LASI survey was administered in 16 languages, and interpretations of the scale items may have differed across languages. Importantly, India-specific scales need to be developed and validated to estimate other forms of discrimination, including structural discrimination, more concretely. It is also essential to define the parameters of structural discrimination in India across various contexts, with a particular focus on government policies at the national, state, district, and subdistrict levels. Notably, a life-course perspective may help understand how ED may be cumulatively shaped by multiple life events across different settings in India, such as schools and workplaces. Emphasis on the life course may better unravel the bidirectional relationship between ED and health status, particularly for middle-aged and older adults^60^. Even though we demonstrate that the state/UT and community levels are relevant to ED, the correlates we include, which were mostly compositional, explain little geographic variation in ED. Future qualitative and quantitative studies must study additional contextual and compositional factors that may drive geographic trends in ED at the household, community, district, and state/UT levels.

## Data Availability

The Longitudinal Ageing Study in India dataset used to support the findings in this study can be accessed at [https://www.iipsindia.ac.in/content/LASI-data](https:/www.iipsindia.ac.in/content/LASI-data) .
